# 52-Year-Old Jehovah’s Witness Female with Weakness

**DOI:** 10.5811/cpcem.2018.3.37699

**Published:** 2018-04-13

**Authors:** Lindsay A. Weiner, George Willis, Zachary D.W. Dezman, Laura J. Bontempo

**Affiliations:** * University of Maryland Medical Center, Baltimore, Maryland; † University of Maryland School of Medicine, Department of Emergency Medicine, Baltimore, Maryland

## CASE PRESENTATION

A 52-year-old woman came to the emergency department (ED) for hemodialysis (HD) due to end-stage renal disease (ESRD). She had spent the preceding several weeks in an outside hospital being treated for pneumonia and bacteremia with daptomycin. She subsequently left against medical advice (AMA) after having her dialysis access catheter removed. She presented to our ED because she needed HD vascular access placement and re-initiation of dialysis treatments. Her medical history included chronic kidney disease secondary to hypertensive nephrosclerosis, anemia, hypertension, deep venous thromboses (DVT), systemic lupus erythematosus, and a seizure disorder of unknown etiology.

The patient, a Jehovah’s Witness, refused blood transfusions; therefore, her anemia was being treated with intravenous (IV) administration of iron, epoetin alfa, and folate. Other medications included nifedipine, clonidine, metoprolol, sevelamer, and prednisone. She was allergic to amoxicillin, azithromycin, hydralazine, labetolol, linezolid, morphine, and vancomycin. She reported previous IV heroin use many years earlier and previous cigarette smoking. She denied alcohol use or any current drug or tobacco use.

Upon arrival, she was alert and in no acute distress with a fever of 38.3° Celsius, heart rate of 87 beats per minute (bpm), blood pressure of 110/65 millimeters mercury (mmHg), respiratory rate of 17 breaths per minute, and pulse oximetry of 99% while breathing room air. She weighed 80 kilograms and was 5 feet 6 inches tall. Her head was normocephalic and atraumatic with moist mucous membranes. Pupils were anicteric, equal, round, and reactive to light and accommodation. The neck was supple and without lymphadenopathy or tenderness. Her lungs had coarse breath sounds with mild bibasilar crackles but no wheezes or rhonchi. There were no retractions or increased work of breathing. Her heart was of regular rate and rhythm without murmurs, rubs, or gallops. Her abdomen was soft with normal bowel sounds and without distention, tenderness, rebound, or guarding. There was no costovertebral angle tenderness. The lower extremities had 1+ pitting edema, 2+ dorsalis pedis pulses, and were without tenderness or deformity. The patient had normal strength and muscle tone throughout all extremities with intact sensation. Old fistulas were present in the left and right upper extremities without palpable thrills. She was alert and oriented to person, place, and time.

**Image 1:**
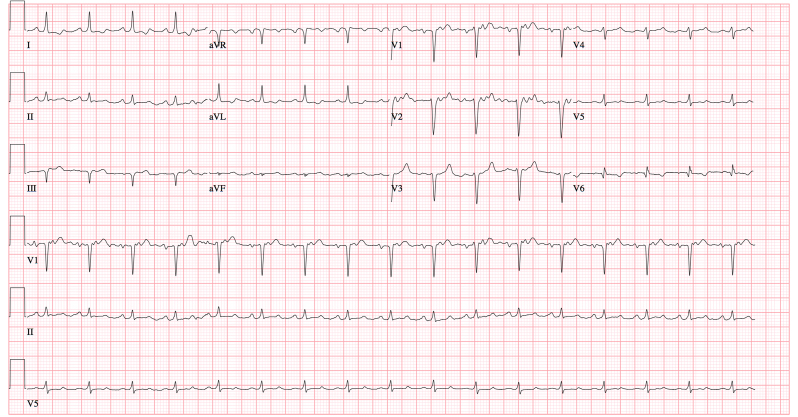
Initial electrocardiogram obtained in the emergency department of a Jehovah’s Witness dialysis patient with serum potassium of 5.8.

The patient’s initial laboratory values are shown in Table 1. These were significant for a slight leukocytosis, anemia to 5.3 g/dL, thrombocytopenia, hyperkalemia, and an elevated creatinine of 11.9 mg/dL. Her initial electrocardiogram (ECG) showed a sinus tachycardia with T-wave inversions in leads I and V6 (Image 1). This was unchanged from a prior ECG. The patient was admitted to the medicine team and was started on daptomycin. While awaiting bed placement, a non-tunneled left femoral dialysis catheter was inserted by the medical intensive care unit (ICU) fellow for initiation of HD. Placement was confirmed by the easy drawback of blood from all ports. During the consent process for catheter placement, it was documented that the patient stated she “would rather die than receive blood transfusions.” Following catheter placement, an attempt was made to start HD, but she became hypotensive and complained of weakness almost immediately. Her vital signs during the initial HD attempt were a blood pressure of 85/56 mmHg with a heart rate of 105 bpm.

**Image 2:**
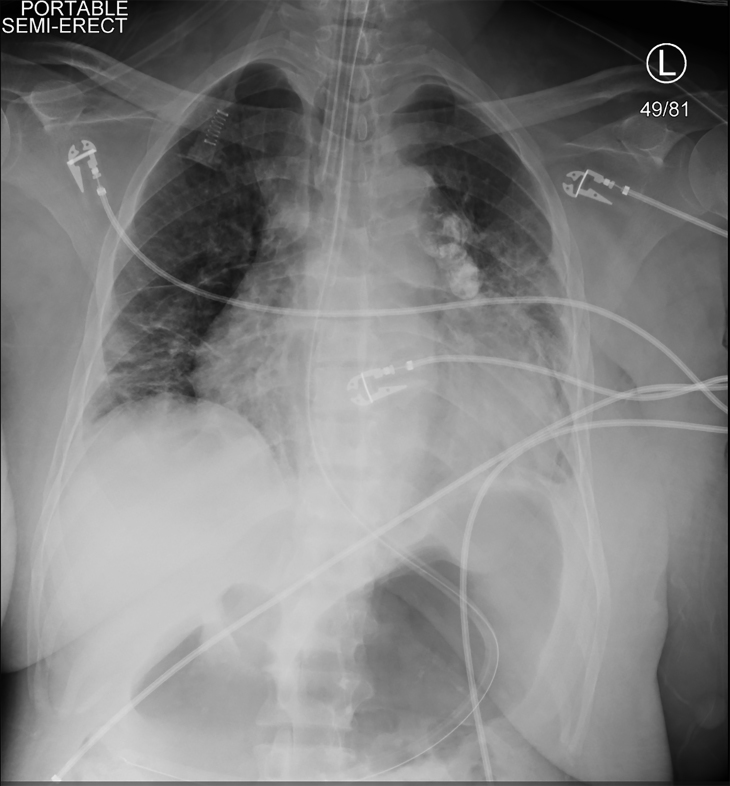
Post-intubation chest radiograph (posteroanterior view) showing proper endotracheal and nasogastric tube placement

Because the patient was symptomatic and hypotensive during HD, continuous renal replacement therapy (CRRT) was initiated. Shortly afterward, she became hypotensive, unresponsive, and had a tonic clonic seizure. Vital signs at that time were a heart rate of 113 bpm, blood pressure 75/48 mmHg, respiratory rate of 20 breaths per minute, and oxygen saturation of 78% on room air. A brief, repeat physical exam showed the patient was unresponsive to painful stimuli and her pupils were equal and reactive. She was given 4 mg of lorazepam and intubated for airway protection. A right arterial femoral line and a right femoral triple lumen venous central line were placed. A chest radiograph confirmed endotracheal tube placement (Image 2). CRRT was halted but the patient remained in shock despite bolus infusions of crystalloid, so vasopressors were started. She was receiving escalating doses of vasopressors when repeat laboratory values indicated a hemoglobin of 1.6 g/dL (Table 2).

Shortly thereafter she went into cardiac arrest with a rhythm of pulseless electrical activity (PEA). Return of spontaneous circulation was achieved after six minutes of cardiopulmonary resuscitation. Increased abdominal distention was noted during the resuscitation; therefore, a pressure-sensing urinary catheter was introduced. Her peak airway and bladder pressures were measured at greater than 60 cmH_2_O and 50 mmHg, respectively. Surgery was then consulted. A massive transfusion protocol was initiated after her next of kin agreed to allow the transfusion of blood products. A focused assessment with sonography for trauma (FAST) was performed, showing free fluid in Morrison’s pouch. As resuscitative measures were continued, an additional study was obtained and a diagnosis was made.

## CASE DISCUSSION

This case presented with a unique and circuitous sequence of events that spiraled very quickly in an unfortunate direction. When presented with patients who have vague or minimal complaints, it is incumbent on providers to search for clues to assist in making the diagnosis. Therefore, I began by searching for the clues in this case.

The patient presented with a chief complaint of needing dialysis. She had recently been admitted at another facility for pneumonia and bacteremia and had her HD catheter removed due to suspicion for line sepsis. She signed out AMA from this facility after receiving daptomycin. It is unknown how long she had been without HD or if she finished her course of antibiotics prior to leaving. However, she is essentially asymptomatic.

Her past medical history is notable for seizures as well as DVTs and anemia. There is no mention of any anticoagulant or antiepileptic medications, which indicates she either did not remember the names of her medications or she was not taking any. Of note, she is a Jehovah’s Witness and therefore received alternate therapies for her anemia, including iron, folate, and epogen.

Her physical exam reveals her to be febrile but with otherwise stable vital signs. I would expect this considering that she had left the other facility and likely didn’t finish her antibiotic course for her pneumonia and bacteremia. Other possibilities include an infection resistant to the antibiotic or a newly-acquired nosocomial infection from her previous hospital stay. She has some coarse breath sounds and bibasilar crackles on her lung exam, which corroborates her pneumonia history. The pneumonia is most likely the source of her fever as well as the bacteremia, which may not present with symptoms.

Her laboratory studies revealed some pertinent findings. She was anemic as well as hyperkalemic. She was also hypocalcemic and hyperphosphatemic. She had some mild uremia as well. Most of these findings would be somewhat expected in an ESRD patient. The anemia may be the result of kidney disease. Other possibilities on the differential diagnosis for anemia include hemorrhage, iron deficiency, and folate deficiency. She does not complain of any blood loss nor do we have the red blood cell indices such as the mean corpuscular volume to be able to make these alternative diagnoses at this point. Her hyperkalemia is most likely from her lack of HD. Her hyperphosphatemia is also most likely from lack of HD; however, daptomycin can cause hyperphosphatemia as well.

While waiting for bed placement, she has HD started during which she begins complaining of feeling weak and is noted to become hypotensive and tachycardic. She then has CRRT initiated and remains hypotensive, becomes hypoxic, unresponsive and has a tonic-clonic seizure requiring intubation and vasopressor initiation. Exam shows equal and reactive pupils in a patient unresponsive to painful stimuli. Laboratory studies reveal a profoundly worsening anemia, worsening hyperkalemia, and interval development of a lactic acidosis. She then goes into a PEA arrest and, after successful return of spontaneous circulation, has blood transfused. However, she is still hypotensive and hypoxic despite intubation. She also has a distended abdomen and peak airway pressures greater than 60 cmH_2_O. A FAST exam reveals free fluid in Morrison’s pouch.

Such a profound status change after a therapeutic intervention requires serious thought. Let’s start with the hypotension and tachycardia during HD and subsequent CRRT. Hypotension during HD is certainly possible with higher flow rates. However, CRRT has much slower flow rates and hypotension is less frequent when CRRT is used. So why is this person in shock requiring vasopressors after initiation of HD?

When approaching patients with shock, the mnemonic SHOCK (septic/spinal; hypovolemic; obstructive; cardiogenic; anaphylactic – K as in lactic) comes to my mind. Sepsis is certainly possible, given her history of bacteremia. However, it is less likely to be the cause of such a profound decompensation, especially with an acute onset in the setting of initiating HD. Hypovolemic or hemorrhagic shock is another possibility. She does have risk factors for both as she may be on anticoagulation with her history of DVT, as well as possibly having uremic platelet malfunction. This will need to be investigated further. Obstructive shock as in pulmonary embolism or tamponade is another possibility. She certainly has risk factors for this with her history of DVT and unknown anticoagulation status. She is also hypoxic, which is not explained by the pneumonia because her original saturations were above 95%. She could also have developed tamponade from uremic pericarditis, although this is less likely given that her blood urea nitrogen (BUN) is not significantly elevated and she had no complaints of pericarditis symptoms. Another possible cause of hemodynamic collapse leading to obstructive shock is an air embolism from the new HD catheter and subsequent initiation of HD through it. Because this is possible, we need to tease this out a little more as well.

Cardiogenic shock is less likely as she is unlikely to have developed a painless myocardial infarction so severe that she went into cardiogenic shock. Anaphylactic and endocrine issues are other possible causes of shock. However, there were no medications administered or any mention of rash or airway compromise to suggest anaphylaxis as a cause. Endocrine shock should be considered as she is on prednisone, making adrenal insufficiency a possible cause. The hyperkalemia and hyponatremia that you would expect with adrenal insufficiency are present; however, there is no mention of noncompliance or vomiting of the steroids, making this possibility less likely.

Seizure during HD is another entity present in this case. She has a history of seizures, and no antiepileptic is on her medication list. So, her seizure could certainly be from noncompliance. Uremic encephalopathy can present as seizures at any point; however, the BUN is only slightly elevated, making this unlikely. Dialysis disequilibrium syndrome is another cause of seizure during HD. However, it usually occurs during the very first HD session and is associated with a rapid drop in BUN. This patient has been on HD for some time and the BUN is not markedly elevated, making this diagnosis unlikely. Lastly, decreased perfusion to the brain from hypotension during HD can induce seizures. The patient became hypotensive during HD, which can happen due to higher flow rates. She was then placed on CRRT, which uses a slower flow rate and has a much lower incidence of hypotension; however, she became more hypotensive. This suggests that there most likely is some other reason for the hypotension rather than simply the HD.

She also has free fluid on her FAST exam and now has a distended abdomen. Coupled with the profoundly worsening anemia, I have to assume she is bleeding into her abdomen. There is nothing other than hemorrhage and hemolysis that could explain such a profound drop in her hemoglobin, and there is no evidence to be suspicious of hemolysis in her history, physical exam, or ancillary studies.

The patient is also experiencing elevated airway pressures on the ventilator. There are several reasons why her airway pressures could be elevated; but in the setting of a distended abdomen and hypotension, abdominal compartment syndrome (ACS) comes to the forefront of my differential diagnoses. Therefore, I have to assume this is a relatively brisk bleed as she has dropped her hemoglobin significantly, gone into PEA arrest, improved with blood transfusions, and is requiring multiple vasopressors to maintain her blood pressure. But why is she bleeding in her abdomen? She has not experienced any trauma to her abdomen unless it happened during the seizure in the hospital; however, if this had been the case I would assume that this history would have been given. She certainly could have experienced some sort of aortic catastrophe such as a ruptured abdominal aortic aneurysm, but it would be very odd for this to have happened during HD.

Now I had to take a step back and view the diagnostic clues as a whole and put these puzzle pieces together. In taking this whole patient presentation into view, one common theme arises in just about every aspect of her sequence of events. Hemorrhage. Anemia? Hemorrhage. Her anemia worsens? Definitely hemorrhage. Hypotension? Hemorrhage. Seizure? Hypoperfusion coming from hypotension coming from hemorrhage. Hypoxia? Profound anemia, which comes from hemorrhage. ACS? Hemorrhage. Lactic acidosis? Hypotension and anemia from hemorrhage.

Now we just have to figure out where this hemorrhage is coming from. When the patient first presented she appeared remarkably stable. She didn’t have any complaints and her vital signs, other than her fever, were within normal limits. She had a HD catheter placed without any mention of complication. She begins HD and her blood pressure drops, her heart rate increases, and she becomes symptomatic nearly immediately. I could easily blame HD as the cause; however, her providers initiate CRRT and her hypotension worsens, making HD less likely to be the cause. Therefore, something has happened prior to the initiation of HD, as she was stable up until HD began. The only thing that happened prior to the initiation of HD was the placement of the HD catheter. There is no mention of any complications or of significant blood loss during the placement. However, it is the only event between the patient being stable and being unstable.

If there was no mention of an obvious complication, what could have happened? Remember that the presumed source of the bleeding is in the abdomen. The HD catheter was placed in the groin, which is not far from the abdomen and retroperitoneal space. If the provider placing the line penetrated the other side of the vessel wall, he/she could easily have placed the HD catheter so that its tip is in the retroperitoneum. Were that the case, the patient would extravasate blood into the retroperitoneal space causing hypotension, decreased perfusion, lactic acidosis, and profound anemia. Once the retroperitoneal space filled up, there would be increased intra-abdominal pressure leading eventually to ACS. A review of the literature reveals a case report of this type of complication.^1^

Therefore, the diagnostic study I would perform is a computed tomography (CT) angiogram of the abdomen and pelvis. This would show where the HD catheter was placed and examine the retroperitoneum for signs of active bleeding.

## CASE OUTCOME

The diagnostic study was a CT angiogram of the abdomen and pelvis. The left femoral HD catheter had punctured the patient’s left common iliac vein and formed a right-sided retroperitoneal hematoma. The surgical team assessed the patient and performed a temporizing bedside laparotomy to treat her acute ACS. This revealed a large, right-sided retroperitoneal hematoma and a large amount of ascites. The patient’s hemodynamics stabilized after this procedure and she was able to come off all vasopressors. She was then transported directly to the operating room with the vascular surgery team for an emergent repair of her left common iliac vein.

She continued to receive multiple blood transfusions during the repair. Afterward she was transferred to the surgical ICU with an open abdomen that was subsequently repaired. The patient woke up completely neurologically intact and was discharged home five weeks after her injury.

## RESIDENT DISCUSSION

ACS is the end point of a spectrum that begins with intra-abdominal hypertension (IAH) and ends when the intra-abdominal pressure exceeds 20 mmHg and is accompanied by end organ dysfunction.^2^ Normal abdominal pressure, even in critically ill patients, should be <12 mmHg. IAH, which can be graded in stages, is defined as a pressure of 12–25 mmHg without the presence of organ dysfunction.

There are several distinct types of ACS. Primary ACS occurs in trauma or surgical catastrophes that result in a large hemorrhagic event, as in the presented case.^3^ Secondary ACS occurs when ICU patients are aggressively resuscitated with large volumes of crystalloids.^4^,^5^ Less common secondary causes include large, space-occupying lesions, bowel or retroperitoneal edema, and pancreatitis. Secondary ACS is insidious and commonly is diagnosed after a delay, further complicating an already high mortality.^4^,^5^

The lethality of ACS is due to compromised organ perfusion and later organ failure. In the kidneys, decreased renal perfusion leads to congestion and oliguria, while increasing compartment pressure causes direct compression of the organ.^6^,^7^ Increased splanchnic vascular resistance results in bowel edema, translocation of gut bacteria, hyperlactemia, and sepsis.^6^,^7^ Cardiac preload may be decreased due to compression of the inferior vena cava causing decreased cardiac output, while right ventricular afterload increases.^7^ Oxygenation is impaired as the diaphragm is pushed cephalad and functional residual capacity decreases.^7^ Intracranial pressure also rises as a result of jugular venous compression impairing venous return. Clinically, the patient will present with hypotension, hypoxia, acute kidney injury, altered mental status, seizures, or unresponsiveness; eventually, the patient experiences total cardiovascular collapse.^6^–^8^

The gold standard for diagnosis of ACS is measurement of bladder pressure indirectly with a urinary catheter.^9^ To measure an accurate pressure, the patient must be in the supine position and the measurement taken at end expiration, with the transducer zeroed and in line with the iliac crest. This measurement has been shown to correlate with directly measured intra-abdominal pressure.^10^ Measurement of bladder pressures every 4–6 hours is adequate in critically ill patients at risk of developing ACS.

The mainstay of treatment for ACS is decompressive laparotomy, usually requiring leaving the patient with an open abdomen post-operatively.^11^ Other secondary temporizing measures include sedation and analgesia, neuromuscular paralysis, decompression of the bowel contents with a naso- or endogastric tube, diuretics, and CRRT.^11^,^12^

The mortality of ACS remains high despite medical intervention. While decompressive laparotomy effectively immediately reduces intra-abdominal pressure, this procedure is associated with multiple medical comorbidities with a mortality that still remains up to 50%.^13^ This may be due to the fact that at the time of decompressive laparotomy, patients are critically ill and the procedure is considered a last resort.

A second major issue for discussion in this case is the ethical dilemma faced by the providers caring for this critically ill patient whose religious beliefs were in opposition to the standard treatment (blood product transfusion) for her disease. The patient had initial documentation in her chart of the physician-patient conversation during which she stated her conscious refusal of blood products. However, she did not have written, signed documentation of blood product-transfusion refusal in the chart. While the patient was unconscious and in extremis, her husband, who is not a practicing Jehovah’s Witness, reversed her decision in an attempt to save her life. Where does this decision fall in the balance of autonomy vs. the power of the next of kin to decide what medical interventions a patient should receive? What happens when the next of kin makes a decision that is against the patient’s choice? In life-or-death situations with ethical dilemmas,where quick decision-making will decide patient outcome, how can this burden be placed on loved ones?

The ethical dilemma, therefore, lies in the idea that while transfusion violates the value of autonomy, it simultaneously fulfills the ethical concept of beneficence. In general, patient personal directives should take precedence over any decisions made by the medical proxy. However, the decision of the family to transfuse on behalf of the unconscious patient, as seen in the current case, is legally appropriate even though it violates the patient’s autonomy.

The ethics committee was consulted after the patient had already received a massive transfusion of blood products and was continuing to receive further red blood cell transfusions. The committee recommended that the patient no longer receive blood product transfusions despite next-of-kin consent. The patient woke up completely neurologically intact and was informed of the blood transfusions. She decided that in the future she would only accept blood bank plasma and family-donated red blood cells during an acute event. The patient was discharged home five weeks after her iatrogenic injury.

## FINAL DIAGNOSIS

Iatrogenic left common iliac vein perforation with resulting abdominal compartment syndrome.

## KEY TEACHING POINTS

1. ACS occurs when the intra-abdominal pressure is above 20 mmHg and end organ damage is present.
2. Primary ACS is caused by trauma or surgery, but be mindful of secondary ACS seen in massive resuscitation, especially in ICUs.
3. The gold standard of ACS diagnosis is a measured bladder pressure.
4. Treatment is a laparotomy as well as temporizing measures such as evacuation of bowel contents and/or sedation and paralysis.

Documented patient informed consent and/or Institutional Review Board approval has been obtained and filed for publication of this case report.

## Figures and Tables

**Table 1:** Initial laboratory results for patient presenting for hemodialysis due to end-stage renal disease.

	Values
Complete blood cell count	
White blood cell count	12.4 K/mcl
Hemoglobin	5.3 g/dL
Hematocrit	15.8%
Platelets	133 K/mcl
Basic metabolic panel	
Sodium	135 mmol/L
Potassium	5.8 mmol/L
Chloride	99 mmol/L
Bicarbonate	27 mmol/L
Blood urea nitrogen	56 mg/dL
Creatinine	11.9 mg/dL
Glucose	189 mg/dL
Calcium	7.2 mg/dL

**Table 2:** Repeat laboratory values.

	Values (prior value)
Complete blood cell count	
White blood cell count	13.4 K/mcl (12.4 K/mcl)
Hemoglobin	1.6 g/dL (5.3 g/dL)
Hematocrit	5.7% (15.8%)
Platelets	167 K/mcl (133K/mcl)
Complete metabolic panel	
Sodium	131 mmol/L
Potassium	6.7 mmol/L
Chloride	99 mmol/L
Bicarbonate	14 mmol/L
Blood urea nitrogen	56 mg/dL
Creatinine	11.9 mg/dL
Glucose	169 mg/dL
Alanine aminotransferase	23 u/L
Aspartate aminotransferase	31 u/L
Alkaline phosphatase	145 u/L
Total bilirubin	0.4 mg/dL
Total protein	5.8 g/dL
Albumin	3.2 g/dL
Lactate	15 u/L
Arterial blood gas	
pH	7.23
PCO_2_	28 mmHg
PO_2_	214 mmHg
